# Validation of the *Spanish Short Self-Regulation Questionnaire (SSSRQ)* through Rasch Analysis

**DOI:** 10.3389/fpsyg.2017.00276

**Published:** 2017-03-01

**Authors:** Angélica Garzón Umerenkova, Jesús de la Fuente Arias, José Manuel Martínez-Vicente, Lucía Zapata Sevillano, Mari Carmen Pichardo, Ana Belén García-Berbén

**Affiliations:** ^1^School of Psychology, Fundación Universitaria Konrad LorenzBogotá, Colombia; ^2^Department of Psychology, School of Psychology, University of AlmeríaAlmería, Spain; ^3^Department of Psychology, Universidad Autónoma de ChileSantiago, Chile; ^4^Department of Education, Education and CareCardiff, UK; ^5^Department of Educational and Evolutionary Psychology, University of GranadaGranada, Spain

**Keywords:** self-regulation questionnaire, Rasch Model, validity, self-regulation measurement, university students

## Abstract

**Background:** The aim of the study was to psychometrically characterize the *Spanish Short Self-Regulation Questionnaire* (SSSRQ) through Rasch analysis.

**Materials and Methods:** 831 Spaniard university students (262 men), between 17 and 39 years of age and ranging from the first to the 5th year of studies, completed the SSSRQ questionnaire. Confirmatory factor analysis (CFA) was carried out in order to establish structural adequacy. Afterward, by means of the Rasch model, a study of each sub scale was conducted to test for dimensionality, fit of the sample questions, functionality of the response categories, reliability and estimation of Differential Item Functioning by gender and course.

**Results:** The four sub-scales comply with the unidimensionality criteria, the questions are in line with the model, the response categories operate properly and the reliability of the sample is acceptable. Nonetheless, the test could benefit from the inclusion of additional items of both high and low difficulty in order to increase construct validity, discrimination and reliability for the respondents. Several items with differences in gender and course were also identified.

**Discussion:** The results evidence the need and adequacy of this complementary psychometric analysis strategy, in relation to the CFA to enhance the instrument.

## Introduction

*Self-regulation* is a psychological construct that has acquired research relevance given its verified relation with the individuals’ capacity to improve their health, well-being and academic, personal and professional success (Karoly et al., 2005; Cañadas-de la Fuente et al., 2014; Clark et al., 2014). There are several conceptual and structural definitions of the construct in each context, which range from a wider vision (*General Self-Regulation)* (Howell and Buro, 2011), to a specific one, that of academic learning (*Self-Regulated Learning)* (Paulino and Da Silva, 2016).

There are various theories on self-regulation, however, there is consensus that it includes skills or conducts such as planning; cognitive, and metacognitive aspects such as self-tracking and motivational aspects such as setting goals. As an example, self-regulation of the learning process can be defined as the degree to which the individuals’ in a behavioral, metacognitive and motivational manner, are active participants of their own learning process (Zimmerman, 1996), which implies skill, consciousness and will.

Despite the many approaches of the study of self-regulation, authors such as Karoly et al. (2005, p. 307) report the existence of common components and point out that “the integration of the science and practice of self-regulation would take a giant step forward if we were to seek greater clarification of our meanings and standardization of our measurement and treatment procedures”.

### Measures of General Self-Regulation

From research that has tried to better comprehend the substance abuse and addictions, Brown (1998) conceptualized the general self-regulation of the behavior of the individuals’ in relation to their specific capacity to plan and manage their conduct in a flexible manner. Subsequently, Brown et al. (1999) developed the *Self-Regulation Questionnaire* (SRQ), which has been adapted to many academic and clinical contexts, thereby obtaining several factorial solutions and items. These solutions can range from the 63 items and seven dimensions of the original questionnaire to 1 dimension and 31 items in the abbreviated version by Carey et al. (2004). In its Spanish adaptation (Pichardo et al., 2014), in order to obtain a more parsimonious measure, an abbreviated version was used (*Spanish Short Self-Regulation Questionnaire, SSSRQ*) with 17 items and four dimensions, with adequate psychometric characteristics for the instrument. Confirmation of the model for this abbreviated version was performed at the item level by reducing the number of items and factors that were most independent from each other. The four-factor model and 17 items were evaluated using Exploratory Factor Analysis (EFA) Finally, the modified model was evaluated using Confirmatory Factor Analysis (CFA). Results were satisfactory, with fit indexes greater than 0.90 and errors around 0.05. Likewise, the Akaike information criterion (AIC index) showed a higher value in the modified model, using the confirmatory sample, than in the initial model with the exploratory sample. The internal consistency was acceptable for the total questionnaire items (α = 0.86) and for the factors: Goal Setting (α = 0.79), Decision Making (α = 0.72) and Learning from Mistakes (α = 0.72). However, the Perseverance factor (α = 0.63) showed low consistency. Finally, correlations between the original version and the version evaluated with the Spanish sample were *r* = 0.85, *p* < 0.001 for the abbreviated version. A study of the relations between Self-regulation (SR), Self-regulation learning (SRL), and the grade received for a class subject was carried out with the total sample. Results showed that SR is significantly related to SRL (*r* = 0.40, *p* < 0.01) and to grade (*r* = 0.15, *p <* 0.01).

The psychometric properties of the SRQ have been previously studied in its short version by means of the *classic test theory*, thus obtaining adequate reliability values and a coherent factorial structure, although with different factorial solutions dependent on the culture or contexts on which the study is carried out. However, the psychometric characteristics of the complete or abbreviated questionnaire have not yet been studied by means of other statistical models such as Rasch analysis, in order to explore its scope and limitations considering the measuring scale, its construct validity and the differential functioning of its items. Amongst the advantages of the *Rasch Model* (RM) versus the *Classic Test Theory* (CTT) are, the possibility to estimate the extent to which each test or item measures the ability of the participants, the joint estimations of the parameters for respondents and items, the invariance of the parameters obtained in different samples, the construction of an interval scale and the possibility of a functioning analysis in terms of order and measure discrimination along the attribute (Bond and Fox, 2012; Arias et al., 2013).

### The Rasch Model as Complementary Strategy of Psychometric Analysis

Namely, the Rasch model is defined by means of testing data against a measuring model and evaluating the degree in which the data fits the model expectations for the construction of the measurement (Smith, 2012). The Rasch analysis involves the probability calculations of a particular person giving a particular answer to a particular question. The Logits scale is a representation of the respondent’s ability to answer the test items with a varying degree of difficulty (Bond and Fox, 2012).

The Rasch model is founded on the principles of unidimensionality and local independence. This *unidimensionality* allows for estimation of the existence of a principal instrument factor and its measurement can be evidenced by means of different statistical estimations that support the validity of the construct, for example, the variance explained by the measure or eigenvalues of the first contrast by means of the Rasch Principal Components Analysis of Residuals (PCAR) (Linacre, 2006; Bond and Fox, 2012). This analysis looks for patterns on the Unexplained Raw Variance and also for groups of items that share the same pattern to identify if they are measuring a common attribute that could represent a secondary dimension. The PCAR is different to the Common-Factor Analysis and so should not be confused with it. *Local independence* indicates that someone’s answer to a question is independent of his or her answer to another question. It is calculated using the expected probability according to the difficulty of each item and to the person’s ability; the procedure compares patterns in every answer, item by item and person by person. The statistics of adjustment are the criteria of the quadratic means (MNSQ) to identify the weight or value of the information (infit) and the sensitivity of the extremes (outfit). The MNSQ values may go from zero to infinite with an expected value of 1. Point-measure (PTMEA) correlation values were also taken into consideration in both items and respondents, which indicate the alignment between the question and the respondent’s ability. Higher values are best and low or negative values can be questionable (Linacre, 2012) as they can indicate a malfunctioning item.

As for *reliability*, the internal consistency of the test under the Rasch analysis is established using the PCAR. It provides estimations both for people and items; the criterion employed in this investigation to evaluate their coefficient was ≥0.80 (Linacre, 2012). In the Rasch model the interpretation of reliability is similar to Cronbach’s alpha in classical test theory (CTT). Another indicator of reliability is the measure of separation indicated by the number of levels in units of standard error, in which the sample of items and individuals can be grouped. The estimation of separation indicates the number of levels from 0.0 to infinity in which the distribution of people or items can be distinguished (Bond and Fox, 2012).

### Objectives

This research seeks to provide an analysis of the psychometric properties of the SSSRQ using Rasch analysis to check the dimensionality, the fit of the items to the model, the functioning of the measurement scale, the construct validity, the reliability and the differential item functioning (DIF) by gender, for each of the four dimensions of the test.

## Materials and Methods

### Participants

The participants were 831 college students, 262 men (31%) and 569 women (68%). With ages between 17 and 39 years, with a mean of 20.7 (SD 4.4.); 552 students of the University of Almeria (66%) and 279 from the University of Granada (33%), both in Spain. As for program distribution, 365 students were of the field of Psychology, (44%); 103 dual degree students of Primary Education and Teaching (12%); 290 elementary school student teachers (35%); 42 students of Sciences of Physical Activity and Sport (5%) and 31 students of the Bachelor program in Psychology (4%). Students were enrolled between the first and fifth year, 665 participants (80%) belonged to the first and second year.

### Instruments

#### *Spanish Short Self-Regulation Questionnaire* (Pichardo et al., 2014)

The questionnaire used for this research was the abridged version of the original questionnaire of self-regulation by Brown et al. (1999), developed by the authors to measure self-regulation of behavior in general, which is defined as what leads people to plan and flexibly address their own behavior according to the demands of the environment through a series of learned strategies (Brown, 1998). In its adaptation to Spanish (Pichardo et al., 2014) a reduced version of 17-items with a 5-point Likert scale, which ranged from 1 (none) to 5 (a lot) and four dimensions was obtained by both exploratory and CFA (see Annex 1).

**FIGURE 1:**
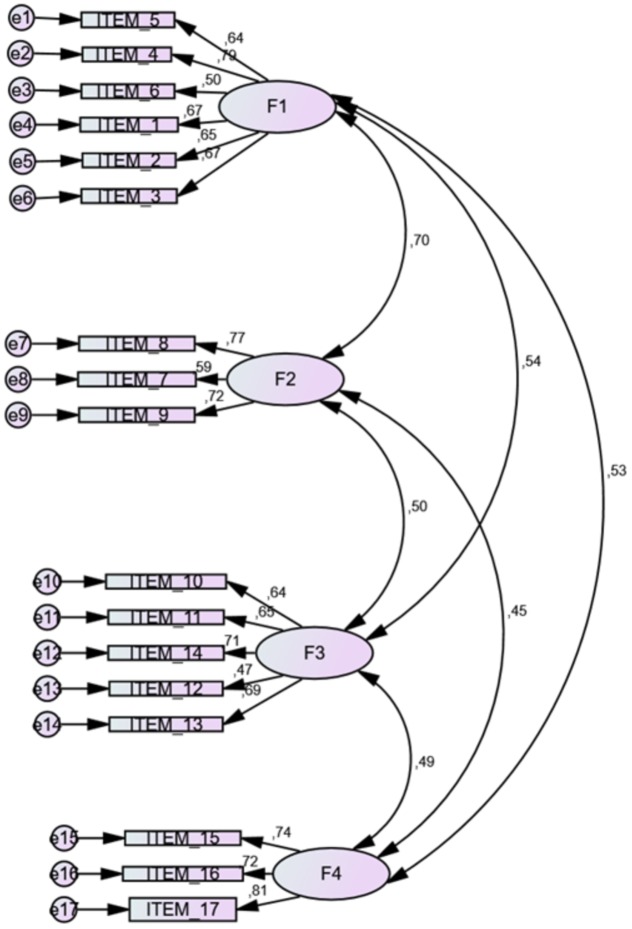
**Dimensions of the Confirmatory Structural Model in the *Spanish Short Self-Regulation Questionnaire* (SSSRQ) version for this sample**.

The CFA performed for this sample showed also the four dimensions (see **Figure 1**), reported by the authors, with adequate general values (all indexes are greater than 0.90), although with a better fit for the case of the female students. See **Table 1**.

**Table 1:** Confirmatory Factor Analysis (CFA) values in this sample.

	Chi-square	Degreesof freedom	NFI	RFI	TLI	IFI	CFI	RMS	HOELT.0.01
G	641.209	113 (*p* < 0.001)	0.974	0.949	0.994	0.972	0.992	0.075	196
M	316.405	113 (*p <* 0.001)	0.931	0.996	0.984	0.959	0.952	0.083	125
F	456.008	113 (*p <* 0.001)	0.968	0.941	0.975	0.997	0.996	0.073	188


*G, general model; M, masculine model; F, feminine model.*

The four dimensions found were: *Goal setting* (F1: 6 items, e.g., “I set goals for myself and keep track of my progress”) *Perseverance* (F2: 3 items, e.g., “I have a lot of willpower”), *Decision-Making* (F3: 5 items, e.g., “I have trouble making up my mind about things”) and *Learning from mistakes* (F4: 3 items, e.g., “I don’t seem to learn from my mistakes”). *A total reliability* value was obtained with a *Cronbach alpha correlation* = 0.87 and for each of the four dimensions as follows: F1 = 0.81, F2 = 0.71, F3 = 0.76, and F4 = 0.79. The psychometric evidence has established that a small number of items (3 items) may be a threat to the validity and reliability of the construct, since the potential variance explained by three items would be small. However, when the *factor loading* value is consistent (in all cases they are greater than 0.70) and the reliability of the factor is adequate, the construction can be accepted as valid. In addition, it should be noted that the *factor loading* with the construct is not the same with an EFA as with a confirmatory analysis, since the latter assumes a more robust methodology. Also, the number of items has been inserted as a limitation, but from the point of view of the measure a smaller number of items is an improvement if the explained variance is the same with fewer items. In the case of the optimal ratio of number of indicators per factor, there seems broad agreement that at least three indicators per factor are desirable (Bollen, 1989), especially under some circumstances, i.e., factor loading >0.6, same size >400 (Gagne and Hancock, 2006). See Annex 1.

### Procedure

The test application was carried out in IT classrooms by means of a web-based platform created for the research. The participants accessed it through a link and a password and answered the questions. There was not a time limit and at all times a researcher was present to solve questions. The students participated voluntarily in the study and signed a written consent prior to participation. The protocols were approved by the Committee on Bioethics in Human Research (University of Almería), and Human Research Ethics Committee (University of Granada) that oversaw the project, and all met the requirements of the Code of Ethics in Psychology and the Spanish Data Protection Act.

### Data Analysis

#### CFA and Reliability

A CFA was conducted in this sample to evidence the factorial validity and to ensure the previous structural adequacy of the inventory. Model fit was assessed by first examining the chi-square to degrees of freedom ratio as well as the *Comparative Fit Index* (CFI) and *Normed Fit Index* (NFI), *Incremental Fit Index* (IFI), and *Relative Fit Index* (RFI). Ideally, these should be greater than 0.90. We also used the *Hoelter Index* to determine adequacy of sample size (Tabachnick and Fidell, 2001). AMOS (v.22) statistical software was used, and the calculation of reliability (Cronbach Alpha) was performed by means of the SPSS (v.22) software.

##### Rasch analysis

An analysis was conducted using the Rasch model to procure the calibration of the SSSRQ items using Winsteps (v. 3.72.3) statistical package. In order to establish the psychometric properties of each of the subscales the following analyses were performed, first, an analysis of the dimensionality of each subscale in order to check for the expected unidimensionality by means of the Rasch PCAR; secondly, a verification of the fit of each of the questions to the model, taking into account the mean squares parameters (MNSQ) using the Rating Scale Model; third, the verification of the adequacy of the response categories to establish the functioning of their order and the Likert scale discrimination; fourth, the reliability for persons and items; and fifth, the invariance of the measure throughout subgroups by gender and course estimating the DIF.

## Results

### Dimensionality

According to the PCAR analysis, it can be concluded that the test only measures a dimension when the variance explained by the measure is ≥40% (Linacre, 2006). Likewise, the first contrast of residuals is also analyzed and must be less than two eigenvalues, so as to rule out the possibility of a second dimension (Smith, 2012). **Table 2** shows the results of the analysis of dimensionality for each subscale. The four subscales that comprise the test (Setting goals, Perseverance, Decision Making and Learning from mistakes) have a single dimension, since they have values above 40% of the variance explained by the measure and none has values higher than 2 in the first contrast.

**Table 2:** Variance of standardized residuals for each subscale.

	Eigen-values	Observed (%)	Expected (%)
**Goal setting**			
Total raw variance =	12.26	100	100
Raw variance as explained by measures =	6.26	51.1	50.6
Unexplained raw variance in first contrast =	1.97	16.1	32.9
**Perseverance**			
Total raw variance =	7.87	100	100
Raw variance as explained by measures =	4.87	61.9	61.7
Unexplained raw variance in first contrast =	1.81	23.0	60.3
**Decision making**			
Total Raw Variance =	10.66	100	100
Raw variance as explained by measures =	5.66	53.1	52.8
Unexplained raw variance in first contrast =	1.11	13.5	28.9
**Learning from mistakes**			
Total raw variance =	10.19	100	100
Raw variance as explained by measures =	7.19	70.6	70.5
Unexplained raw variance in first contrast =	1.68	16.5	56


*Similar values are expected in the observed and expected raw variance percentages.*

### Fit Indexes

The Rasch model is based on the premise that the persons that have a higher skill will answer correctly to all of the easy elements, meanwhile, the individuals that do not have the same skill level are not expected to answer correctly even the easiest items (Bond and Fox, 2012).

As criteria of Rasch model fit, MNSQ values of Infit and Outfit between 0.5 and 1.5 were considered (Bond and Fox, 2012). Values greater than 1.5 indicate that the item is erratic and values lower than 0.5 indicate that the item is very predictable. According to the results (see **Table 3**) none of the items obtains values outside of the established range, hence, all of the items show acceptable Rasch model fit. Moreover, the mean values for persons are also within the model fit parameters (in **Table 3** see last two rows by subscale).

**Table 3:** Infit and outfit estimation and reliability for each item by factor.

			INFIT		OUTFIT		PT-MEASURED		RELIABILITY	
						
Item	Measure	Model SE	MNSQ	ZSTD	MNSQ	ZSTD	CORR.	EXP.	Persons	Items
Goals									0.79	0.96

2	-0.06	0.06	1.28	5.0	1.23	4.2	0.72	0.71		
6	-0.47	0.06	1.21	3.8	1.21	3.7	0.61	0.69		
3	0.23	0.06	1.11	2.2	1.13	2.4	0.75	0.72		
1	0.25	0.06	0.90	-1.6	0.92	-1.6	0.71	0.72		
5	-0.30	0.06	0.82	-3.5	0.83	-3.5	0.70	0.70		
4	0.36	0.06	0.63	-8.4	0.63	-8.4	0.80	0.73		

Item mean	0	0.06	0.99	-0.5	0.99	-0.5				
*SD*	0.31	0	0.23	4.6	0.22	4.5				
Person mean	1.6	0.69	0.98	-0.2	0.99	-0.2				
*SD*	1.65	0.11	0.94	1.4	0.95	1.4				

Perseverance									0.75	0.98

7	0.13	0.06	1.33	6.1	1.31	5.9	0.75	0.81		
8	-0.48	0.06	0.87	2.7	0.86	-3.0	0.84	0.81		
9	0.35	0.06	0.78	-4.9	0.78	-4.9	0.84	0.81		

Item mean	0	0.06	0.99	-0.5	0.98	-0.7				
*SD*	0.35	0	0.24	4.7	0.24	4.7				

Person mean	0.80	0.96	0.98	-0.3	0.98	-0.3				
*SD*	2.04	0.07	1.24	1.4	1.23	1.4				

Decision making									0.77	0.98

12	-0.50	0.05	1.38	6.9	1.38	6.9	0.61	0.70		
10	0.58	0.05	0.99	-0.2	1.00	0.0	0.73	0.73		
11	-0.29	0.05	0.89	-2.3	0.88	-2.6	0.74	0.71		
14	0.01	0.05	0.88	-2.5	0.88	-2.6	0.75	0.72		
13	0.20	0.05	0.84	-3.5	0.86	-3.1	0.75	0.72		

Item mean	0	0.05	1.00	-0.3	1.00	-0.3				
*SD*	0.38	0	0.2	3.8	0.2	3.8				

Person mean	0.42	0.63	0.99	-0.1	1.00	-0.1				
*SD*	1.42	0.09	0.81	1.3	0.82	1.3				

Learning from mistakes									0.80	0.99

15	-0.31	0.06	1.22	3.9	1.20	3.6	0.84	0.85		
16	1.17	0.06	1.00	0.0	0.98	-0.4	0.87	0.87		
17	-0.86	0.07	0.77	-4.8	0.76	-4.9	0.87	0.84		

Item mean	0	0.06	1.00	0.2	0.98	-0.6				
*SD*	0.86	0	0.18	2.5	0.18	-3.5				

Person mean	1.22	1.04	0.97	-0.3	0.96	-0.3				
*SD*	2.46	0.11	1.41	1.3	1.41	1.2				


*MNSQ (infit and outfit) values between 0.5 and 1.5 in each item are considered to fit the model. The items are put in hierarchies by dimension, in order of difficulty. The last two rows “Person mean” are a summary of the values for persons; for this analysis, the extreme values (zero and perfect scores) are eliminated.*

Moreover, there are no negative correlations between the items and the measurement (see **Table 3**, column PT-Measure-CORR) and the correlation values tend to be moderate to high being 0.61 the lowest value for items 6 and 12; which means there is a correct alignment between the question and the ability of the respondent; likewise, the correlation values are very close to the expectations of the model (see **Table 3**, column PT-Measure-EXP).

### Reliability

**Table 3** (“Reliability” column) shows the reliability values for respondents and items for each of the analyzed factors, being in the four subscales high for the items and low to moderate for respondents. In addition, the separation index for persons by subscale is presented: *Setting goals* (1.96); *Perseverance* (1.67), *Decision Making* (1.81) and *Learning from mistakes* (1.99). A low index of separation is considered for persons, when values are lower than 2, which was evidenced in the four subscales. It indicates that the instrument is not sensitive enough to identify people with high and low proficiency in the measured variable (Smith, 2012). The separation values for the items were: Setting goals (4.94); Perseverance (5.5), Decision Making (7.47), and Learning from mistakes (12.76). Values below 3 are considered low for the items, as opposed to the results presented in the four subscales. This indicates that the sample is large enough to confirm the hierarchy of item difficulty, namely the construct validity of the instrument (Smith, 2012).

### Estimation and Interpretation of b Parameter

The Rasch model analyzes the construct validity based on the hierarchy of the items; it is calculated and shown on the map of items by difficulty estimates thereof. Items must form a continous “pattern” where low difficulty items are located at the bottom, those of moderate difficulty in the middle, and high difficulty ones are located at the top. The map shows the distribution of the items to the right and that of the respondents to the left.

**FIGURE 2:**
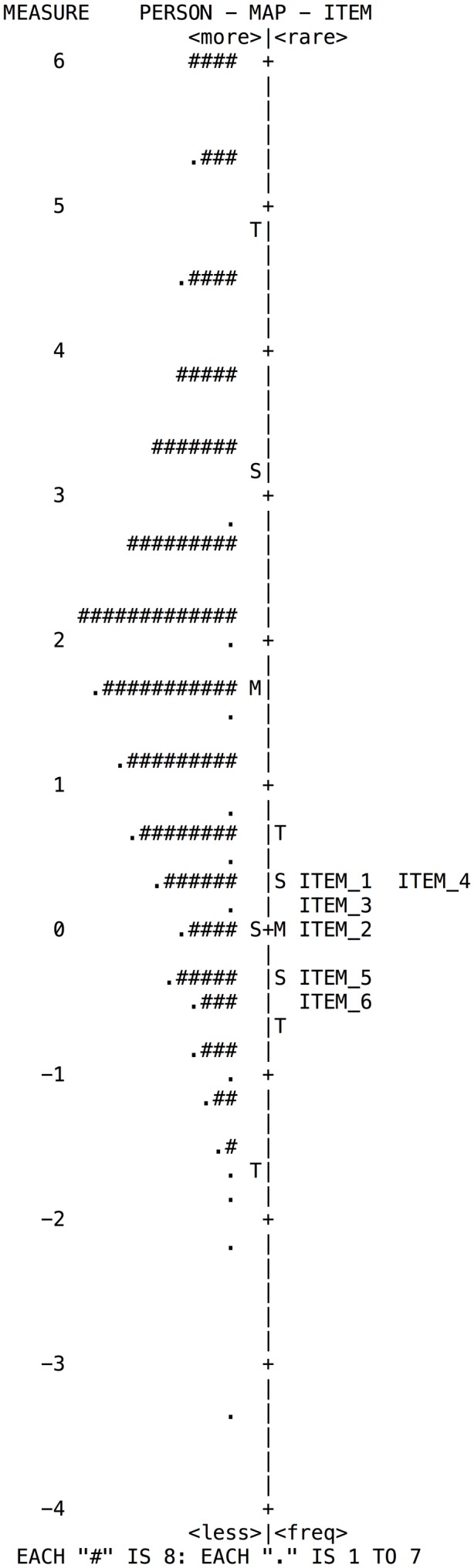
**Wright map of person and items for the Goal Setting subscale**.

**FIGURE 3:**
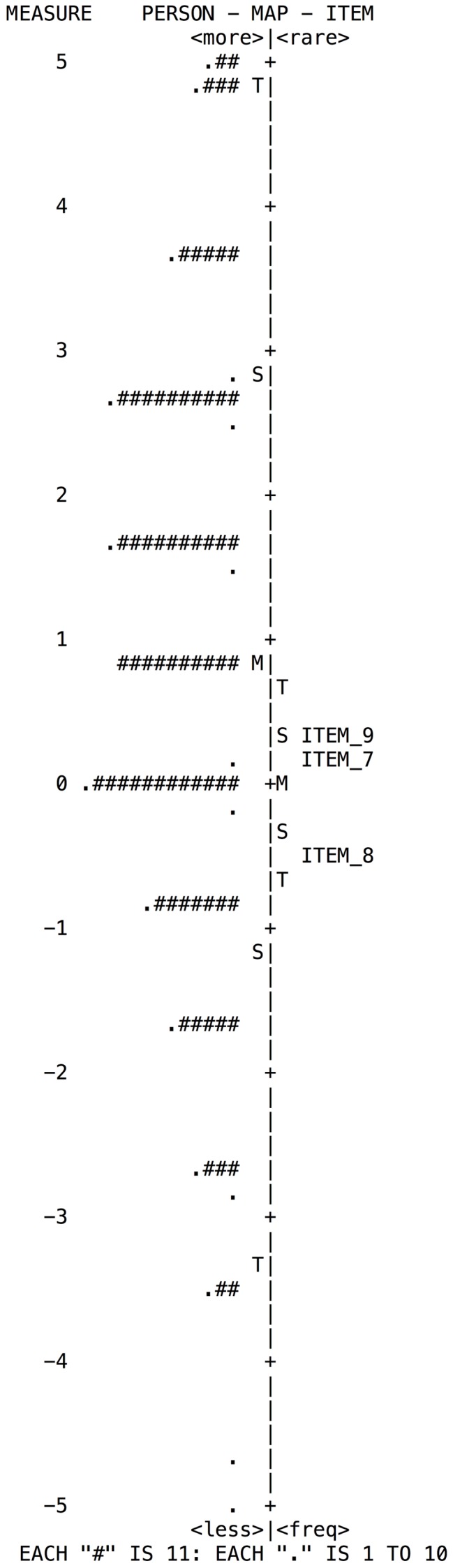
**Wright map of person and items for the Perseverance subscale**.

**FIGURE 4:**
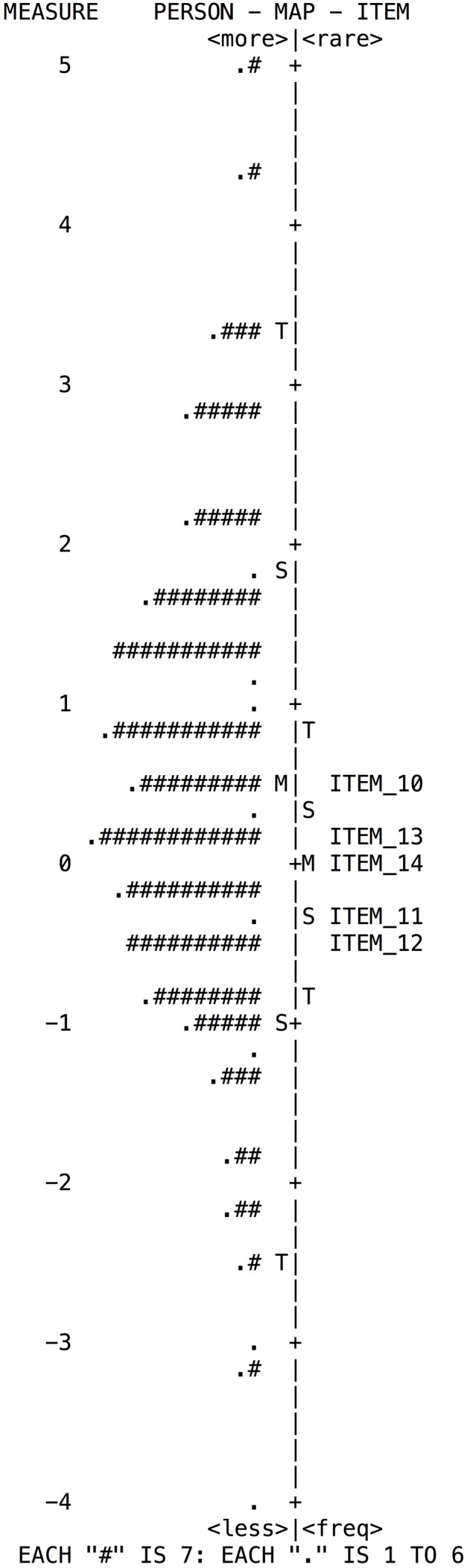
**Wright map of person and items for the Decision-Making subscale**.

**FIGURE 5:**
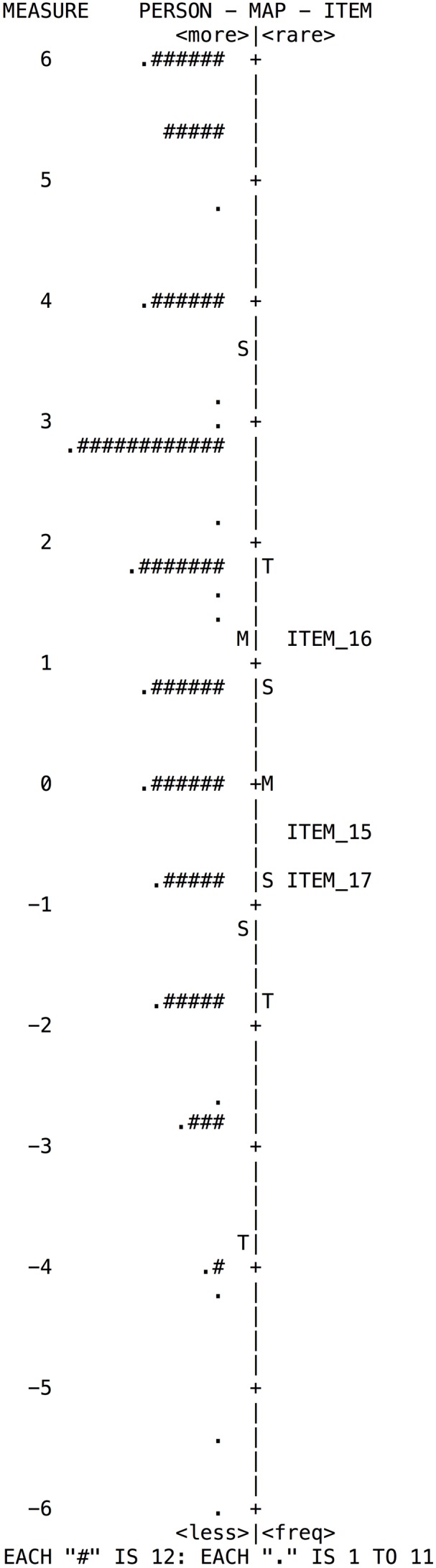
**Wright map of person and items for the Learning from mistakes subscale**.

According to the distribution maps presented by the subscales (**Figures 2**–**5**); despite there is appropriate distribution of the items, these are insufficient to cover the range of ability of individuals, mainly in the high range. For example, in the subscale *Goal Setting* (**Figure 2**) there is a lack of questions to cover the skill level of individuals in the medium and high range.

### Functioning of the Response Categories

**FIGURE 6:**
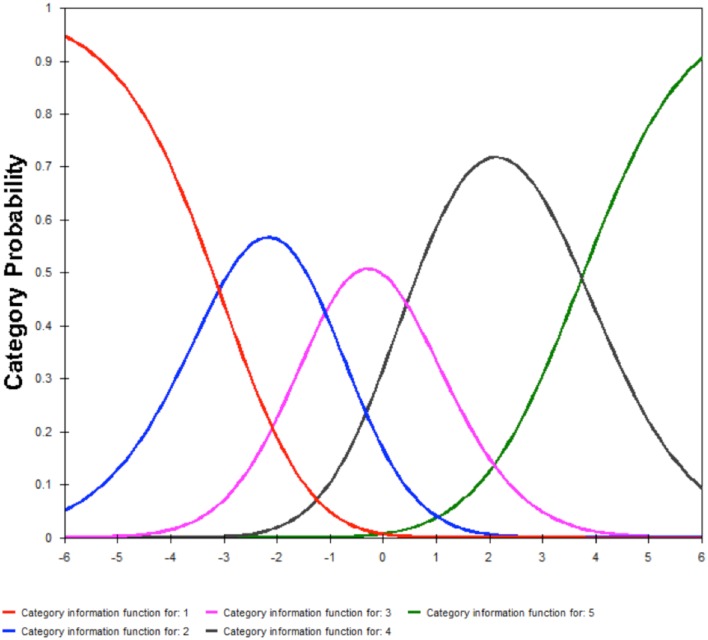
**Probability curves for the five response categories**.

The response categories of the test are: (1) nothing, (2) a little, (3) fairly, (4) usually, and (5) a lot. Using the Rating Scale Model (RSM) for polytomous items, the order of the categories and the discrimination of each one was verified, namely, the clear differentiation of each of the values. The four subscales showed a correct order – none of them had an inverted order. Moreover, by means of the *Probability Curves of Categories*, the differentiation of each of the categories along the attribute measurements was found (1 to 5) (See **Figure 6**).

### Differential Functioning of Items

Differential Item Functioning was used to assess the measurement invariance across subgroups between men and women (see **Table 3**) and within courses (see **Table 4**). DIF exists when a group of people that is tested does not have the same probability of answering correctly to an item even if they have the same level of the measured variable. The criteria assumed in order to rule out DIF were, in one hand, that values were below 0.5 logits, which is the difference in the difficulty of an item between the two groups, and in the other hand, that the t values were less than 2 and no significant differences were observed (*p* ≤ 0.001) (Bond and Fox, 2012). Values were included in the report of **Tables 4**,**5**.

**Table 4:** Analytical summary of the gender – based differential behavior of items.

		DIF Contrast		
		
Item	Subscale the item belongs to	Men	*t*-value	*p*
(12) I have so many projects I find it hard to concentrate on one	Decision making	0.60	5.71	0.000


*Differential Item Functioning Contrast values indicate that the item has more difficulty for men.*

**Table 5:** Analytical summary of course-based differential behavior of items.

Item	Subscale	Courses	DIF Contrast	*t*	*p*-value
2	Goals	1–4	0.56	-3.24	0.001
6	Goals	5–1	2.39	2.72	0.034
5	Goals	2–5	1.55	-2.46	0.049
6	Goals	5–2	2.29	2.61	0.040
6	Goals	5–3	2.74	2.83	0.018
6	Goals	5–4	2.29	2.59	0.041
7	Perseverance	4–3	1.01	2.12	0.046
12	Decision-making	5–1	2.34	3.09	0.021
13	Decision-making	1–5	1.67	-3.25	0.011
12	Decision-making	5–2	2.55	3.37	0.020
13	Decision-making	2–5	1.54	-3.01	0.019
13	Decision-making	3–5	1.68	-2.72	0.016
12	Decision-making	5–4	2.12	2.76	0.013
13	Decision-making	4–5	1.38	-2.66	0.028


*Under the Courses column, the first number corresponds to the course year for which the item is easier.*

Evidence of DIF by gender was found on item 12 of the subscale Decision Making, being it more difficult for men. Likewise, DIF was found by courses on subscale Setting goals for items 2, 5, and 6. Being particularly relevant for item 6, which is easier for fifth year students than for students from first to fourth year. The DIF contrast values were superior to 0.5, but the significance test is above (*p* ≤ 0.001), reason for which this items need to be further investigated. In regard to the Perseverance subscale, only item 7 showed some DIF evidence, being it easier for fourth year students than for third year students. Finally, the subscale Decision Making, two items showed some DIF evidence on the reverse items 12 and 13. Item 12 is easier for fifth year students than for first, second and fourth year students. Item 13 is more difficult for fifth year students than for students from first through fourth years.

## Discussion

The results of the Rasch analysis for the SSSRQ (Pichardo et al., 2014) indicate that the objective raised in this research was relevant: to assess the differential contributions of the Rasch analysis in relation to the use of *CFA*. In this analysis, the data confirm a factorial invariance for *gender* and a similar fit of the final four-factor confirmatory model for university students. This assumes that the construct has a similar structure for both in college students.

As indicators of the global fit the MNSQ values of Infit and Outfit between 0.5 and 1.5 were used for person and items. It was found that the mean for persons and items are within the model fit parameters. However, the results showed that the test would benefit from the inclusion of items of greater difficulty for a broader cover in this personal meta-skill throughout the four subscales. In addition, the subscales Perseverance and Learning from Mistakes would also benefit from the incorporation of items of lesser difficulty. Considering the above, greater construct validity would be obtained. From the Rasch model, a normal distribution in people and a correspondence between items and people of about 80% (Smith, 2012) is expected. When this ratio is insufficient, it is advisable to add more questions to cover all skill levels of people (low, medium or high) according to the distribution on the map. This result is related to the low level of separation obtained between people in the four subscales, which translates to a low level of reliability for people. Although the reliability of the SSSRQ for the measure is very adequate, the reliability values decrease for people, because the instrument is not sensitive enough to detect or discriminate people with low and high level of self-regulation. For the purposes of differentiating people with different levels of self-regulation in the four subscales, it would be necessary to improve the sensitivity of the test.

Otherwise, when analyzing the differential behavior of items (DIF), one of the 17 items had differential functioning in favor of women, with moderate values. The DIF analysis between courses indicates greater relevance in the values found in the DIF contrast, which is interpreted as an effect size and comes to attention when it is greater than 0.5. Is important to clarify that items with values above *p* ≤ 0.001 on the significance test were included in **Table 4** mainly because the test showed significant t values. The t test is more sensitive to the sample size and in future research should be revised if these values indicate a possible differential behavior of the items with reduced DIF values. However, since the existence of DIF does not necessarily imply a bias in the item (Wright and Stone, 1999) all the listed items must be studied further in order to determine whether a gender or course bias actually exists. Particularly for item 6 (“If I make a resolution to change something, I pay a lot of attention to how I’m doing it”) which content seems to relate to the capacity of self tracking and items 12 (“I have so many plans that it’s hard for me to focus on any one of them”) and 13 (“When it comes to deciding about a change, I feel overwhelmed by the choice”), that are both reverse items. Item 12 content is related with the focus capacity in planning, which seems to be easier for fifth year students. Item 13 content is related with the easiness of making decisions without feeling overwhelmed, which seems to be more difficult for fifth year students.

In short, both the dimensionality of the subscales and the items fit to the model are adequate. Similarly, the functioning of the measurement scale was confirmed and so indicates that the values of the Likert scale used are correct (ordered) and suitably work along the measurement for each subscale. A background of analyses based on the Rasch model for the SSSRQ version was nonexistent. The results in this research show evidence of the need and appropriateness of this complementary strategy of psychometric analysis due to the fact that it allows detection of possible improvements for the instrument such as: an increase its construct validity, its discrimination capabilities among individuals’ with different self-regulation levels or the differential functioning of some items.

However, the results presented in this study have several limitations. On the one hand, the sample comprises only college students in three study areas. This suggests that it would be necessary to include a more diverse sample in regard to age, occupation, education level, and even distinctive psychosomatic features. Since this SSSRQ version is designed to measure overall self-regulation in different contexts – not just academia – including a more diverse sample could lead to studying the behavior of the test in other areas. Future research should corroborate if the limitation of this instrument to determine the medium and high levels of general self-regulation, could be compensated by means of cluster analysis. Previous investigations have allowed the establishment of diverse relationships of inter-dependence between low-medium-high self-regulation subjects in regard to variables like the coping strategies and self-regulated learning (de la Fuente et al., 2015a), the learning approach and the academic performance or the academic confidence (de la Fuente et al., 2015b). Moreover, it would be important to study the psychometric characterization in other Spanish-speaking countries to contrast the transcultural validity of the instrument and its possible uses outside Spain.

## Ethics Statement

All subjects gave written informed consent in accordance with the Declaration of Helsinki. The protocols were approved by the Committee on Bioethics in Human Research (University of Almería) and Human Research Ethics Committee (University of Granada) which managed the project and all met the requirements of the Code of Ethics in Psychology and the Spanish Data Protection Act.

## Author Contributions

AGU: Final writing, review research, analysis of data. JF: Coordination R&D Project, data collect, final writing, analysis of data. JM: Review research, data collect. LZ: Review research, analysis of data, final writing. MP: Review research, analysis of data, final writing. ABG: Data collect, review research.

## Conflict of Interest Statement

The authors declare that the research was conducted in the absence of any commercial or financial relationships that could be construed as a potential conflict of interest.
