# Supplementary material for: Validation of the Spanish Short Self-Regulation Questionnaire (SSSRQ) through Rasch Analysis
Source: Front Psychol. 2017 Mar 1;8:276. doi: 10.3389/fpsyg.2017.00276 (PMC5331067; doi:10.3389/fpsyg.2017.00276)
Supplement: Supplementary file 1 [file Data_Sheet_1.docx]

ANEX 1. Factor (F); original numbering and content of the 17 items from confirmatory analysis

| Factor | Item. | Content |
| --- | --- | --- |
| Goal setting | 1. | I usually keep track of my progress toward my goals. |
|  | 2. | I have a hard time setting goals for myself (-). |
|  | 3. | I have trouble making plans to help me reach my goals (-). |
|  | 4. | I set goals for myself and keep track of my progress. |
|  | 5. | Once I have a goal, I can usually plan how to reach it. |
|  | 6. | If I make a resolution to change something, I pay a lot of attention to how I’m doing. |
| Perseverance | 7. | I get easily distracted from my plans (-). |
|  | 8. | I have a lot of willpower. |
|  | 9. | I am able to resist temptation. |
| Decision making | 10. | I have trouble making up my mind about things (-). |
|  | 11. | I put off making decisions (-). |
|  | 12. | I have so many plans that it’s hard for me to focus on any one of them (-). |
|  | 13. | When it comes to deciding about a change, I feel overwhelmed by the choice (-). |
|  | 14 | Little problems or distractions throw me off course (-). |
| Learning from mistakes | 15. | I don’t seem to learn from my mistakes (-). |
|  | 16. | I usually only have to make a mistake one time in order to learn from it. |
|  | 17. | I learn from my mistakes. |
